# Deubiquitylation of deubiquitylases

**DOI:** 10.1098/rsob.170016

**Published:** 2017-06-28

**Authors:** Saba Haq, Suresh Ramakrishna

**Affiliations:** 1 Department of Life Science, College of Natural Sciences, Hanyang University, Seoul, South Korea; 2 Graduate School of Biomedical Science and Engineering, Hanyang University, Seoul, South Korea; 3 College of Medicine, Hanyang University, Seoul, South Korea

**Keywords:** auto-regulation, deubiquitylating DUBs, proteasome degradation, self-deubiquitylation, ubiquitylation

## Abstract

Deubiquitylating enzymes (DUBs) reverse the ubiquitylation of target proteins, thereby regulating diverse cellular functions. In contrast to the plethora of research being conducted on the ability of DUBs to counter the degradation of cellular proteins or auto-ubiquitylated E3 ligases, very little is known about the mechanisms of DUB regulation. In this review paper, we summarize a novel possible mechanism of DUB deubiquitylation by other DUBs. The available data suggest the need for further experiments to validate and characterize this notion of ‘Dubbing DUBs’. The current studies indicate that the idea of deubiquitylation of DUBs by other DUBs is still in its infancy. Nevertheless, future research holds the promise of validation of this concept.

## 1. Introduction

The ubiquitin proteasome pathway, prominently responsible for the targeted degradation of usually short-lived proteins (e.g. cell-cycle regulatory proteins), is a major part of various cellular regulation events. Two decades ago, Ye *et al*. uncovered three enzymes required for this process and termed them ubiquitin activating enzyme (E1), ubiquitin conjugating enzyme (E2) and ubiquitin ligase (E3) [1]. E1 activates ubiquitin by producing a ubiquitin-adenylate intermediate, followed by subsequent transfer of ubiquitin to an active site cysteine residue. Next, a thioester bond is formed between ubiquitin and the cysteine on E2, which results in their conjugation. In the final step, the E3 ligase not only assists E2 in recognizing the protein substrates, but also catalyses the transfer of ubiquitin from the E2 cysteine to a lysine residue on the target protein. Ubiquitin can bind singly (monoubiquitin) or in chains (polyubiquitin) on the target protein, ultimately governing the fate of the target protein. Interestingly, lysine-48 ubiquitin chains have been shown to play a crucial role in ATP-dependent proteasomal degradation [2], whereas K63-linked chains are heavily involved in the modification of protein location, interaction and function [3]. In summary, the series of processes termed ‘ubiquitylation’ has varied and vital influences on protein degradation through the proteasome and lysosome pathways [2], activation and inactivation of proteins [4], protein localization [5] and the modulation of protein–protein interactions [6].

## 2. Deubiquitylases and their classification

**Figure 1.**
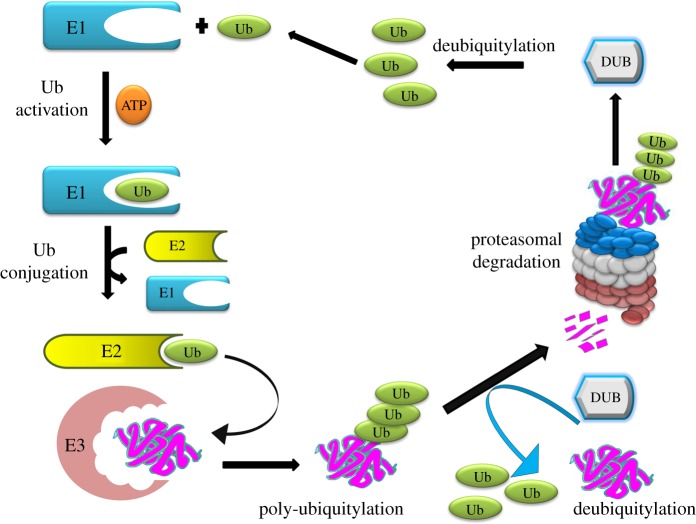
The ubiquitin proteasome system. Ubiquitylation occurs through the subsequent actions of E1 (ubiquitin activating enzyme), E2 (ubiquitin conjugating enzyme) and E3 enzymes (ubiquitin ligase) on the mediation of ubiquitin ligation to target proteins. DUBs counteract E3 ligase-mediated ubiquitylation and recycle the ubiquitin molecules for further use.

Deubiquitylases (also referred as deubiquitylating enzymes) (DUBs) are proteases that remove monoubiquitin or polyubiquitin from proteins, thereby leading to the recycling of trapped ubiquitin molecules (figure 1) [7]. Their ability to regulate the fate of subcellular proteins makes them a prominent diagnostic and therapeutic target for research [8]. Additionally, multiple studies have clearly illustrated the auto-ubiquitylation of E3 ligase and the subsequent interaction of DUBs with this auto-ubiquitylated protein [9,10]. DUBs encoded by the human genome have been classified into five families [11]. Among these, the ubiquitin-specific proteases (USP/UBP), the ubiquitin C-terminal hydrolases (UCH), the ovarian tumour (OTU) domain and the Josephin domain are papain-like cysteine proteases. However, the DUBs belonging to the fifth family have a JAB1/MPN/Mov34 metalloenzyme (JAMM) domain and function as zinc-dependent metalloproteases [12].

**Figure 2.**
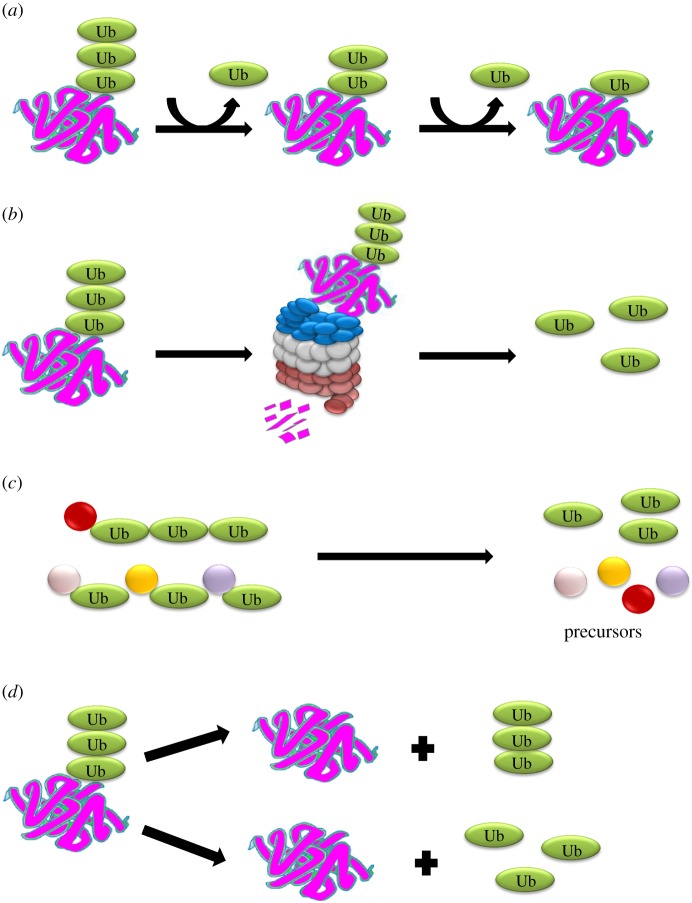
Catalytic roles of DUBs. DUBs contribute to four major cellular events: (*a*) editing of ubiquitin chains, (*b*) recycling of ubiquitin, (*c*) processing of ubiquitin precursors and (*d*) reversal of ubiquitin conjugation.

DUBs play a significant role in various cellular activities including editing ubiquitin chains, recycling ubiquitin molecules during ubiquitylation, processing ubiquitin precursors and reversing ubiquitin conjugation (figure 2) [7,13]. Moreover, DUBs regulate diverse cellular functions such as cell cycle progression, apoptosis [14], DNA repair, prevention of protein degradation, cellular reprogramming [15], chromosome segregation, kinase activation, gene expression, and localization and degradation of signalling intermediates [16–18]. The main feature of deubiquitylating enzymes is that, unlike the ubiquitylation process, which requires a set of three enzymes, DUBs are single enzymes that have the capability to antagonize not only substrate ubiquitylation, but also E3 auto-ubiquitylation. This article primarily focuses on the effects of E3s on DUBs and the possibility of such DUBs to deubiquitylate each other. Scientists working with a particular DUB might imagine by what strategy, how and when will it be rescued, shuttled or subtly shifted from its allotted fate and how this mechanism will further affect other cellular proteins. To the best of our knowledge, this is the first report summarizing the novel concepts of intra- and inter-DUB stabilization and regulation. To clarify this concept, we coin the term ‘Dubbing DUBs’ to describe such deubiquitylating enzymes.

## 3. Ubiquitylation of deubiquitylases

It has been previously reported that DUBs can undergo ubiquitylation [19], and recent studies have focused on DUB-regulation pathways. Numerous studies have demonstrated that DUBs such as AMSH [20], USP7 [21,22], USP15 [23], USP20 [24] and USP5 [25] undergo ubiquitylation. ICP0 is a herpes simplex virus type 1 (HSV-1) regulatory protein that targets USP7 for ubiquitylation and proteasome-mediated degradation, leading to lower cellular USP7 level during HSV-1 infection [21]. The validation of K869 as the ubiquitylation site of USP7 further confirms the interaction of this DUB with E3 ubiquitin ligases [22]. Likewise, USP14, UCHL5, UCHL3, Otub1 and BAP1 are targets of ubiquitylation in HeLa cells [26]. USP4 (i.e. UnpEL) is a substrate for ubiquitylation by the E3 ligase Ro52; however, in the absence of a substrate, Ro52 auto-ubiquitylates*.* Additionally, UnpEL/USP4 can reverse this process by specifically deubiquitylating Ro52 via isopeptidase activity [27]. Ataxin-3 is a deubiquitylating enzyme that serves a role in protein homeostasis maintenance, cytoskeleton regulation, and transcription and degradation of misfolded chaperone substrates. It binds to long polyubiquitin chains of more than four ubiquitins and has the ability to trim them [25]. Studies have confirmed the ubiquitylation of USP7 [28], USP36 [29], DUB-1 [30] and DUB-1A [31], but limited data are available regarding the significance of these modifications. Interestingly, multiple studies have hinted at a possible correlation between the PEST motif and the regulation of DUBs via the proteasomal degradation pathway [29,30]. Also, SUMOyl modifications have been found to decrease USP25 binding and the hydrolysis of polyubiquitin [32]. Additionally, ubiquitylating enzymes (e.g. E3 ligases) undergoing self-ubiquitylation can be rescued by DUBs. Numerous cases have shown this regulation to be compartment-specific and to involve the recruitment of two DUBs: USP7 for the nuclear E3 and USP9X for the cytosolic E3 ligase [33].

**Figure 3.**
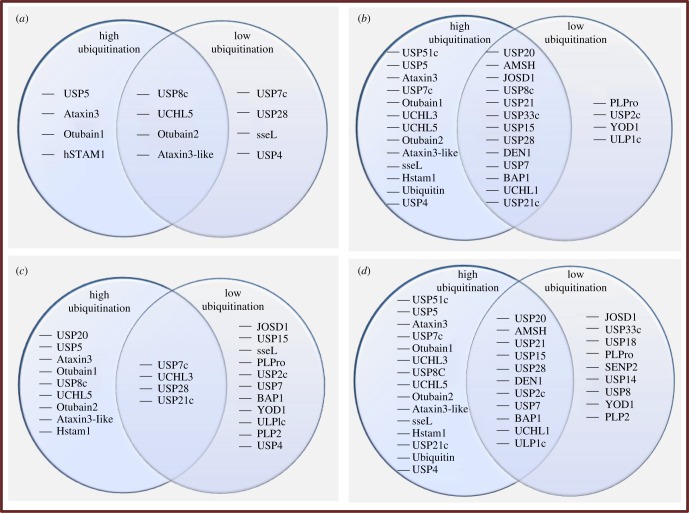
Monoubiquitylation of deubiquitylating enzymes by (*a*) Carp2, (*b*) Praja1, (*c*) Murf1 ubiquitin ligase and (*d*) their mixture (Carp2, Praja1 and Murf1). The circles on the left and right represent high and low ubiquitylating DUBs, respectively. The overlapping area between the two circles represents DUBs that undergo intermediate ubiquitylation. (*a*) Level of ubiquitylation of deubiquitylating enzymes by Carp2 ubiquitin ligase. (*b*) Level of ubiquitylation of deubiquitylating enzymes by Praja1 ubiquitin ligase. (*c*) Level of ubiquitylation of deubiquitylating enzymes by Murf1 ubiquitin ligase. (*d*) Level of ubiquitylation of deubiquitylating enzymes by Carp2, Praja1 and Murf1 ubiquitin ligases. Inferred from Loch & Strickler [34].

Protein microarray experiments have illustrated that DUBs undergo ubiquitylation by various E3 enzymes. DUBs have been shown to bind to a single ubiquitin molecule (monoubiquitylation) or a chain of multiple ubiquitin molecules (polyubiquitylation). Moreover, different E3 enzymes can work additively when they are co-incubated with DUBs *in vitro* (figure 3*a–d*). These DUBs are located in different cellular compartments, and their mono- or polyubiquitylation and deubiquitylation can have a marked influence on different cellular or nuclear proteins and signalling events in that region. Loch & Strickler [34] demonstrated that, upon incubation with DUB substrates, only Praja1 E3 ligase had the capability to exhibit polyubiquitylation of DUBs. Moreover, enhanced polyubiquitylation was seen when Praja1, Carp2 and Murf1 ubiquitin ligases were co-incubated with a DUB substrate immobilized on the microarray. The DUBs that underwent polyubiquitylation include USP51c, USP5, Ataxin-3, USP7c, Otubain1, UCHL3, UCHL5, Otubain2, Atexin-3-like, sseL and hSTAM1. The mono- and polyubiquitylation of DUBs by ubiquitin ligases has been confirmed *in vitro* [9,34]. When mixed ubiquitin ligases (i.e. Carp2, Praja1 and Murf1) were replaced with whole cell lysates, the monoubiquitylation of the substrate increased in a dose-dependent manner [34].

DUBs can undergo mono- and polyubiquitylation, consequently impacting their activity and substrate binding. Ataxin-3, a well-studied deubiquitylating enzyme, undergoes ubiquitylation at the K117 position, resulting in increased catalytic activity of normal as well as disease-related ataxin-3 [35]. Similarly, ubiquitin C-terminal hydrolase L1 (UCHL1) is another deubiquitylating enzyme that undergoes ubiquitylation. Monoubiquitylation of UCH-L1 depends on its ubiquitin binding site that directly binds to the conjugated ubiquitin. This post-translational modification confers limitations to the enzymatic activity of UCH-L1 by preventing its binding to ubiquitylated substrates or free ubiquitin, leading to impaired function. Furthermore, permanent monoubiquitylation abrogates UCH-L1 capacity to regulate free ubiquitin level in the cellular environment [36]. Likewise, BAP1 deubiquitylating enzyme is multi-monoubiquitylated by ubiquitin-conjugating enzyme UBE2O. This monoubiquitylation occurs at the highly conserved nuclear localization signal (NLS) and results in cytoplasmic localization and sequestration of BAP1. Further, deletion of N or C terminal regions of UBE2O has been found to entirely disrupt ubiquitylation of BAP1 [37].

Likewise, USP4 is a DNA repair regulating protein and invokes a model where ubiquitin adducts control its interactions and functions [19]. Similarly, USP6 is another deubiquitylating enzyme found to undergo mono- and polyubiquitylation. The evolutionarily conserved calcium (Ca^2+^)-binding protein calmodulin (CaM), usually regarded as an important transducer of Ca^2+^ signals, has been identified as a novel interactor for USP6. Calmodulin directly interacts with USP6 in a Ca^2+^-dependent pattern (i.e. this binding is regulated by physiological alteration in the level of Ca^2+^ *in vitro* as well as *in vivo*). However, the mutant USP6 having alterations in the CaM binding region leads to significant reduction in ubiquitylation pattern, implying that direct interaction of Ca^2+^/CaM with USP6 has a key role in promoting USP6 monoubiquitylation. A possible hypothesis is that Ca^2+^/CaM promote the monoubiquitylation of USP6 by increasing its binding to ubiquitin ligase. Interestingly, monoubiquitylated USP6 has been demonstrated to retain its capacity for interacting with calmodulin, suggesting that it might have a role in allowing the complex to remain associated with the ubiquitin ligase for chain elongation. On the contrary, polyubiquitylated USP6 is incapable of further binding with CaM, thereby invoking a probable mechanism for the release of ubiquitin ligase [2]. Some other DUBs undergoing ubiquitylation include USP7 [4], USP15 [38], USP19 [6] and USP25 [39]. USP7 undergoes reversible monoubiquitylation, while USP19 undergoes polyubiquitylation that subsequently causes its degradation [4,6].

The USP25 isoform (USP25m) has been found to undergo mono- or polyubiquitylation. The data from mass spectrometry samples show a lysine residue at position 99 (K99), also reported to be a sumoylation target, to be the preferential site for ubiquitylation [32]. The deletion of the ubiquitin binding domains (UBDs) noticably decreases but does not absolutely abolish USP25m ubiquitylation. This suggests that the UBDs of USP25 increase its ubiquitylation, and that this process of ubiquitylation might be occurring in other lysine residues with less efficiency in the deleted mutants. On the other hand, upon deletion of 106 amino acids in the C-terminal region, USP25m undergoes polyubiquitylation and is targeted for proteasome degradation, demonstrating the relevance of the C-terminus region in the stability of the USP25m protein [39]. Cumulatively, these data suggest that DUBs undergo mono- and polyubiquitylation. Thus, ubiquitylation of DUBs has been shown to be an important event for maintaining an optimal level of DUBs for cellular homeostasis. Fundamental questions about the impacts of ubiquitylation on multiple cellular processes remain to be researched. Taken together, these findings indicate that further studies on ubiquitylation of DUBs have to be considered to significantly answer such questions and to gain advanced understanding about the DUB-mediated cellular network.

## 4. Deubiquitylation of deubiquitylases

Unlike ubiquitylation, limited data are available regarding the mechanisms of stabilization and regulation of DUBs. The word ‘cryptic’ has been associated with the regulation of DUB activity due to lack of information on this topic [7]. Although the process of ubiquitin removal from a particular deubiquitylating enzyme by itself or via association with another DUB is less well characterized, this event is equally significant for the maintenance of cellular homeostasis.

Various bioinformatics tools have been used to demonstrate possible interactions between DUBs. For example, CompPASS was used to discover 774 potential DUB-interaction candidates. Validation of interactions between different DUBs by immunoprecipitation (IP) has revealed that DUBs do interact with each other. For example, USP11 has been found to interact with USP4, USP7 and USP15; and PSMD7 interacts with USP15 and PSMD14 [8]. These findings motivate researchers to study the possible consequences of DUB interactions with each another. This information would help in addressing the missing knowledge regarding the DUB regulatory network. However, the nature and possible effects of these aforementioned interactions need to be further researched.

Microarray assemblies have been used to determine the behaviours of deubiquitylating enzymes on multiple DUB substrates that were ubiquitylated by the combined action of Murf1, Carp2 and Praja1 E3 ubiquitin ligases. The loss of ubiquitylation signal indicates potential deubiquitylating enzyme activity. Among the 14 enzymes (USP7, USP15, USP21c, JOSD1, PLP2, AMSH, UCHL5, USP2c, USP8c, USP20, USP28, JOSD2, PLPro and Otub2) selected for testing deubiquitylation, two (USP2c and USP8c) showed significant loss of monoubiquitylation and polyubiquitylation from ubiquitylated DUB substrates.

**Figure 4.**
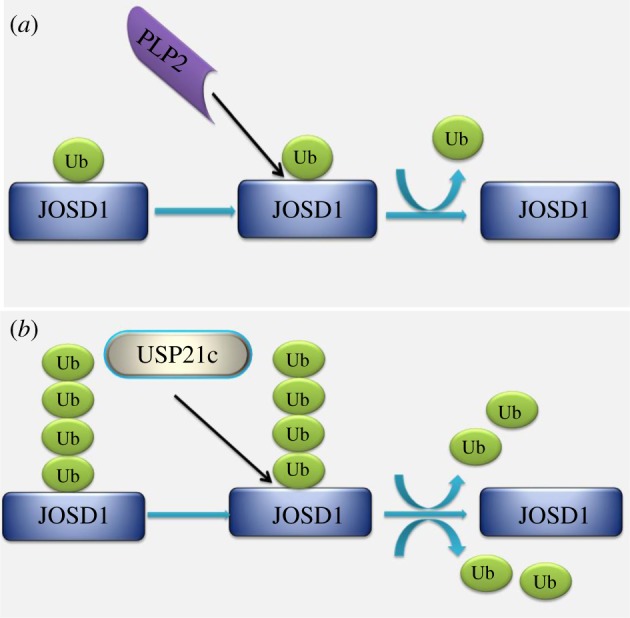
Representations of the microarray data inferred from Loch & Strickler [34]. (*a*) Removal of monoubiquitin from JOSD1 by PLP2 deubiquitylating enzyme. (*b*) The figure illustrates JOSD1 to be a putative substrate of USP21c deubiquitylating enzyme as per its capability to completely remove the ubiquitin chains.

**Figure 5.**
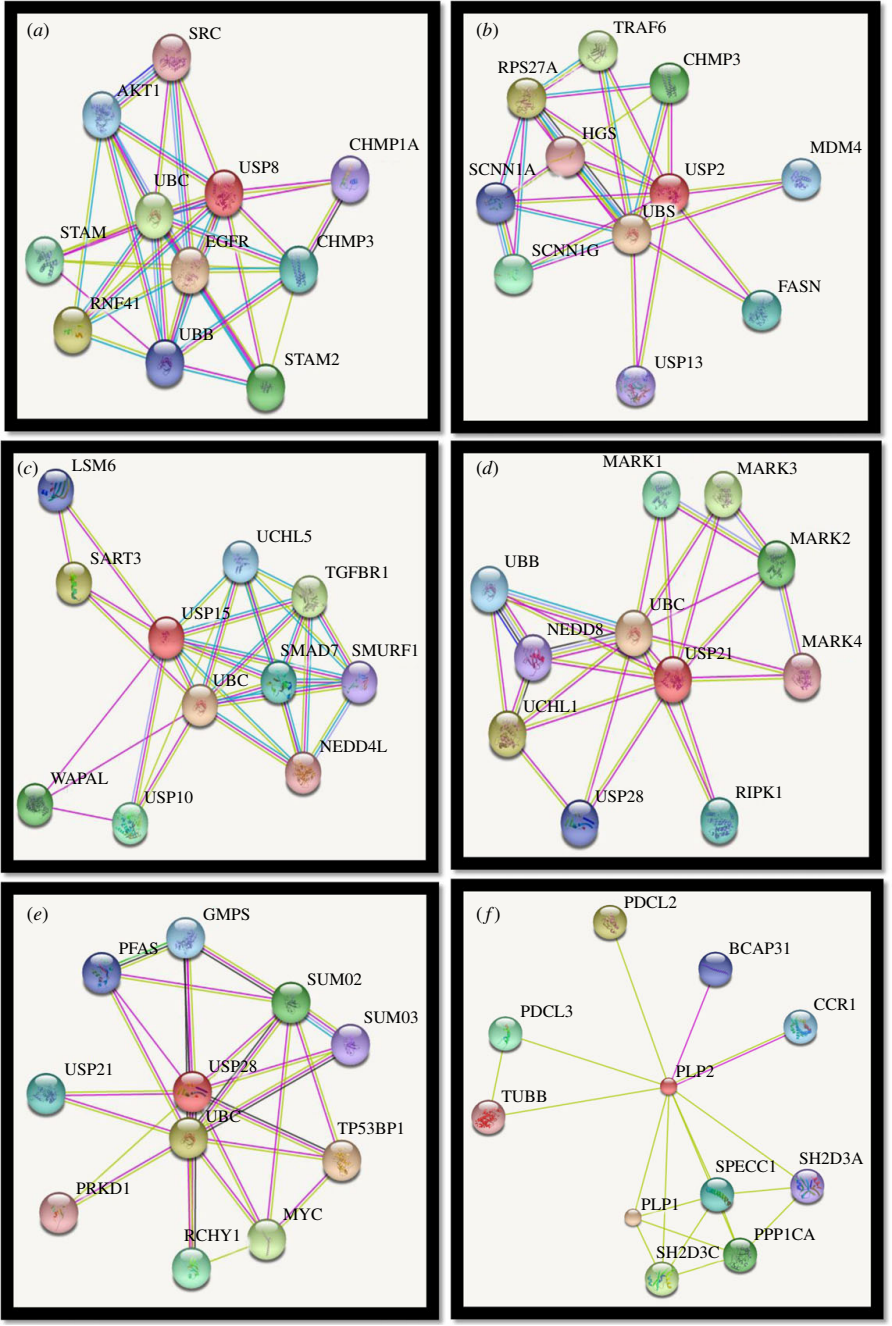
The potential interactors of (*a*) USP2, (*b*) USP8, (*c*) USP15, (*d*) USP21, (*e*) USP28 and (*f*) PLP2 as illustrated in STRING: Functional Protein Association Network software [40].

USP2 and USP8 can be identified as potential ‘universal DUBs’ as their catalytic domains have been found to cause complete deubiquitylation of ubiquitylated DUB substrates. In addition, USP21c has been shown to cause complete removal of ubiquitin chains from not only itself, but also JOSD1 (figure 4*b*). During chain-shortening, USP21c causes removal of the final monoubiquitin, thereby demonstrating an ‘all or none’ response. Some DUBs (e.g. USP15) are incapable of completely deubiquitylating themselves but can cause chain-shortening of JOSD1. PLP2 can remove ubiquitin from JOSD1 (figure 4*a*) and UCHL1 *in vitro,* and USP4 is a putative DUB substrate of PLPro (table 1). Microarray data indicate that DUBs possess the ability to deubiquitylate themselves or other deubiquitylating enzymes; however, ubiquitylation of array-immobilized DUBs might be a technical artefact as this assembly might expose an otherwise inaccessible lysine. Therefore, the DUBs confirmed to undergo ubiquitylation via microarray were further tested. These DUBs have displayed smearing, characteristic of polyubiquitylation, upon probing with tandem ubiquitin-binding entities (TUBEs) in soluble phase *in vitro* ubiquitylation reactions. Polyubiquitylation data obtained in solution, as opposed to monoubiquitylation events observed in microarrays, indicate the possibility that the immobilization of the substrate might have impeded ubiquitin chain formation [34]. By testing the potential binding partners of six deubiquitylating enzymes (USP2, USP8, USP21, USP15, PLP2 and PLPro) via the STRING Bioinformatics tool, we identified the interactions among DUBs (figure 5). However, further investigation is required on the nature and subsequent consequences of these interactions.

**Table 1.** Five DUBs and their potential DUB substrates as confirmed by protein microarray [34].

sr. no.	DUBs	modes of action
1	USP2	the catalytic domain completely deubiquitylates the ubiquitylated DUB substrates
2	USP8	USP8 completely deubiquitylates the ubiquitylated DUB substrates
3	USP21	JOSD1 and USP21c are potential substrates
4	PLP2	JOSD1 and UCHL1 are putative substrates
5	PLPro	USP4 is a putative substrate

Researchers investigated the roles of USP2c, USP21c and PLP2 in deubiquitylating soluble, ubiquitylated JOSD1 and UCHL5 DUB substrates. USP2c and USP21c were able to remove ubiquitin from both UCHL5 and JOSD1, but PLP2 was able to remove ubiquitin from JOSD1 only, suggesting the potential ability of a deubiquitylating enzyme to prevent degradation of other members of the DUB family. Loch & Strickler verified the variable characteristics of DUBs, including the ability to cleave chains in a substrate-specific pattern (e.g. USP15), stringent substrate selection that cleaves in an ‘all or nothing’ manner (e.g. USP21c), and promiscuously cleaving deubiquitylating enzyme (e.g. USP28) and DUBs that selectively remove the final monoubiquitin (e.g. PLPro and PLP2), via a microarray-based assembly [34]. Other studies have validated the occurrence of DUB auto-regulation, as well as regulation by other members of the DUBs family.

Interaction of ubiquitin or ubiquitin-like molecules with numerous protein substrates has been identified as a multi-purpose regulatory mechanism. Likewise, auto/self-deubiquitylation embodies a striking auto-regulation function, contributing to localization, lifespan, or catalytic activity of DUBs [41]. Researchers have suggested that the process of DUB auto-deubiquitylation might pose wider applications and impacts than currently appreciated [37]. Examples of DUBs that undergo deubiquitylation are UCH-L1 [36], BAP1 [37], USP4 [42], USP6 [2], USP7 [4], USP15 [42], USP19 [6] and USP25 [39].

Monoubiquitylation is a cellular regulatory mechanism that can be reversed by deubiquitylation. Monoubiquitylated UCH-L1 undergoes auto-deubiquitylation in an intramolecular fashion only (i.e. within the single molecule of ubiquitylated UCHL1 enzyme). The level of monoubiquitylated UCH-L1 in the cell is dependent on the balance of its ubiquitylation and deubiquitylation events. In contrast, upon monoubiquitylation, the protein generally tends to alter its subcellular localization [43], but monoubiquitylation of UCH-L1 produces no effect on its cellular distribution. Transient UCH-L1 modification might have a role in facilitating protein–protein interactions that would later be regulated via its auto-deubiquitylation. Alternatively, another possible role of monoubiquitylation of UCH-L1 might be its involvement in maintenance of ubiquitin homeostasis. Interaction of UCH-L1 and ubiquitin has been found to enhance monoubiquitin level by guarding ubiquitin from degradation. Thus, this binding might have a role in providing a readily accessible pool of ubiquitin molecules, especially in the case of low cellular ubiquitin concentration. However, the availability of this pool of additional ubiquitin is also dependent on auto-deubiquitylation of UCH-L1 [36].

BAP1 is a deubiquitylating enzyme that acts as a transcriptional regulator for mammalian development as well as a tumour suppressor [44,45]. BAP1 nuclear localization and its catalytic activity are essential for its growth-suppressive features, and it is inactivated or mutated in several cancers [46,47]. Furthermore, depletion of BAP1 through RNAi technology has been found to cause defects in cell-cycle progression, thereby strongly suggesting that it is a master key regulator of cellular proliferation. Monoubiquitylation of BAP1 via E3 ligase activity of UBE2O at its NLS is counteracted by BAP1 auto-deubiquitylation. BAP1 auto-deubiquitylation is dependent on intramolecular interactions. Disruptions in the process of auto-deubiquitylation can lead to improper BAP1 cellular distribution. Cancer-derived BAP1 mutations in the catalytic site, known for abrogating the process of auto-deubiquitylation and promoting its cytoplasmic retention, demonstrate that the BAP1 auto-deubiquitylation event safeguards tumour suppression, suggesting that, in particular, auto-deubiquitylation of BAP1 is a critical event for regulating its cellular proliferation activity [37].

USP4 plays an important role in promoting DNA-end resection and DNA double-strand break (DSB) repair via the process of homologous recombination (HR). It interacts with CtIP and MRE11-RAD50-NBS1 (MRN) complex and regulates CtIP recruitment to the site of DNA damage. The two catalytic sub domains within USP4 (i.e. D1 and D2) are sufficient to mediate these interactions [42]. The wild-type UnpEL/USP4 deubiquitylating enzyme undergoes self-deubiquitylation, but a catalytically inactive mutant fails to do so, thereby strengthening the concept that DUBs can regulate themselves and each other via their deubiquitylating activity [19]. USP4, ubiquitylated on multiple cysteine residues, requires catalytic activity for reversing its ubiquitylation. Ubiquitylation of USP4 interferes with its interactions with the DNA damage response proteins MRN and CtIP, ultimately hampering DNA end resection and abolishing HR. The self-deubiquitylation of USP4 enables it to recruit CtlP to DNA-damage sites, undergo interactions with MRN and CtIP, and eventually cause DNA double-strand break-repair [48].

The USP6/TRE17 is an oncogene that induces tumorigenesis in mice and neoplastic growth in humans. Limited data are available regarding its mechanism of transformation and regulation; however, researchers have speculated about the association of its USP domain with tumorigenesis [1]. Monoubiquitylated USP6 has been reported to promote its own deubiquitylation in vivo; however, it is uncertain whether it can catalyse its own deubiquitylation or requires an intermediate deubiquitylating enzyme [2]. These findings suggest that deubiquitylation of USP6 might have a critical role in tumour progression.

USP7, also referred to as herpes-associated ubiquitin-specific protease (HAUSP), is involved in various cellular processes (e.g. DNA replication, apoptosis and transcriptional regulation). It interacts with and deubiquitylates p53, Hdm2 and Hdmx proteins, and consequently shields the cells from apoptosis. USP7 undergoes monoubiquitylation, which is reversed by auto-deubiquitylation. However, it is unclear if auto-deubiquitylation occurs in an intraspecific or interspecific manner. The impacts of USP7 deubiquitylation and its probable effects on regulating its protein level or catalytic capabilities must be further investigated. However, one can predict that the process of auto-deubiquitylation might possibly assist USP7 in regulating its protein level since treatment with a catalytic inhibitor results in significant reduction of USP7 protein [4]. In abnormal cases, an elevated level of USP7 was reported to promote oncogenesis; therefore, USP7 might be a significant target for therapeutic intervention. Thus, research on the regulation of USP7 is important for the development of novel inhibitors and disease treatment [5].

The structural relatedness of USP15 to USP4 might also play a role in DNA damage repair. Similar to USP4, the interactions between USP15 and its protein target, SMAD2/3 [38], are governed via USP15 self-regulation, while catalytically inactive USP15 lacks the ability to bind to SMAD2/3 protein [42]. Smad proteins are involved in signal transduction through transforming growth factor-beta superfamily ligands, thereby regulating differentiation, death and cell proliferation processes via activation of receptor serine/threonine kinases [49]. We predict that the process of USP15 self-regulation will lead to enhanced stability of its substrates (i.e. SMAD2/3 subsequently affects the cellular process).

Another deubiquitylating enzyme, USP19, associates with itself, removes associated ubiquitin moieties and undergoes self-deubiquitylation. This auto-deubiquitylation activity is involved in the stabilization of USP19, suggesting that the protein stabilization of USP19 is regulated via self-association and intermolecular deubiquitylation. Self-deubiquitylation of USP19 might indirectly affect the levels of cellular inhibitors of apoptosis (c-IAP) as USP19 regulates c-IAP stability, ultimately affecting diverse cellular processes such as apoptosis, NF-κB signalling and oncogenesis [6].

Another example of DUB auto-deubiquitylation is USP25, which encodes three protein isoforms via alternative splicing. Among those, two isoforms are ubiquitously expressed; however, the longest isoform, USP25m, is localized in muscle tissues. Mono- or playubiquitylated USP25 reverses ubiquitylation by catalysing its own deubiquitylation. Moreover, USP25 has been found to undergo dimerization/oligomerization. A plausible explanation for this action is intermolecular auto-deubiquitylation of USP25. The probable influences of USP25 auto-deubiquitylation on its own stability require further validation [39].

In addition to auto-regulation, DUBs that are reported to interact with other DUBs exhibit enhanced protein stability. The USP family is composed of UBL domains located at the amino-terminal, C-terminal or within their catalytic domain. Likewise, USP4 has one UBL domain at the N-terminal of its catalytic domain, and the other one is within its catalytic domain. USP39 binds with USP4 via its UBL domain and correspondingly enhances the stability of USP4 in T cells [46,50], implying that USP39 might be deubiquitylating USP4. However, further study is required to validate this finding. The impacts of USP4 trans-regulation and its implications on T cells need to be elucidated further. Undeniably, rigorous research in this field is required for better understanding of DUBs' manifold roles in control of their own regulation.

In addition to ubiquitylation, various post-translational modifications governing DUB regulation include phosphorylation and SUMOylation*.* Phosphorylation via Akt has been found to relocate nuclear USP4 to the membrane and cytoplasm and maintains its protein stability [51]. USP1 phosphorylation via cyclin-dependent kinases (Cdks) might have a role in prevention of premature degradation of USP1 during cell cycle progression [52]. Furthermore, USP1, as a prototypical deubiquitylating enzyme, must bind with UAF1 for its catalytic action. Its phosphorylation at Ser313 is essential for interacting with UAF1 and subsequent stimulation of USP1 activity [14]. The USP1/UAF1 complex promotes homologous recombination and DNA cross-link repair via deubiquitylation of two critical DNA repair substrates: Fanconi anaemia protein (FANCD2) [53] and proliferating cell nuclear antigen (PCNA) [48,54]. Akt phosphorylates USP14 at the Ser432 residue, resulting in activation of its deubiquitylating activity [55]. Like phosphorylation, SUMO modification regulates DUB activity (e.g. SUMOylation at the N-terminal domain of USP28 has a negative effect on its deubiquitylating action [15]). SUMOylation on Ataxin-3 has been reported to partially enhance its stability but produce no effects on its subcellular localization [35]. Taken together, this information prompts us to ask if all DUBs manifest auto- or trans-deubiquitylation. Moreover, are DUBs that undergo auto-deubiquitylation also subject to trans-deubiquitylation? Finally, are there any ‘master DUB regulators' that can deubiquitylate multiple DUBs? Comprehension derived from research on dubbing DUBs is expected to offer novel insights into the manifold queries on the DUB regulatory network and correspondingly direct the introduction of innovative strategies for molecular therapies against diseases.

## 5. Conclusion and future prospects

In light of the data discussed in our manuscript, an entirely new picture of DUB regulation has emerged. DUBs are critical key players in diverse processes such as (i) spermatogenesis (e.g. USP2, USP8, USP9y, USP14, USP26, Uchl-1, Uchl-3 and CYLD) [52], (ii) cancer biology (e.g. USP7, USP10 and USP11) [44,56,57], and (iii) stemness and differentiation (e.g. USP7, USP9x, USP22, USP44 and Psmd14) [58].

A growing body of evidence supports DUBs as essential for stem cell pluripotency and differentiation [58,59]. USP9X deubiquitylating enzyme is widely expressed in stem cells including neural stem cells (NSCs), preimplantation blastomere embryos [60], neuronal progenitors (NPs), haematopoietic stem cells (HSCs) and adult epidermal stem cells [61]. The enzyme favours self-renewal of neural progenitor cells [60] and drives the differentiation of skeletal muscle stem cells [11]. Similarly, USP22 is essential for embryonic stem cell (ESC) differentiation into three germ layers [12]. USP44 deubiquitylating enzyme is downregulated during the process of ESC differentiation [62], suggesting that ESC differentiation is dependent on an optimal level of USP44 expression. USP7-mediated deubiquitylation hinders the proteasomal degradation of repressor element 1 silencing transcription factor (REST), consequently promoting neural stem and progenitor cells maintenance [13]. Cumulatively, these findings demonstrate the major roles of DUBs in the maintenance of stem cell pluripotency and differentiation, further strengthening the notion that a deubiquitylating enzyme that regulates these DUBs would contribute to stem cell regulation. For instance, a deubiquitylating enzyme that can stabilize USP7 might enhance stemness; in addition, a deubiquitylating enzyme that can deubiquitylate USP22 could promote ESCs differentiation.

DUBs also play vital roles in spermatogenesis (e.g. during gonocyte and spermatogonia development and meiosis regulation [63]), as well as in oogenesis (e.g. oocyte maturation and fertilization [17]). USP2-knockout mice display abnormal aggregation of elongated spermatids, resulting in fertility defects [18,64]. Histological data have shown that USP14-deficient testes display abnormal spermatogenesis [16]. USP26 regulates androgen receptor hormone-mediated spermatogenesis and steroid production [65], suggesting that the DUBs stabilizing USP2, USP14, USP26 and UCHL1 ultimately regulate the processes of spermatogenesis and oogenesis.

Similarly, DUBs have a pivotal role in tumour biology. USP10 and USP11 stabilize p53, a tumour suppressor protein, and thus impede cancer progression [66,67]. Finding a potent deubiquitylating enzyme to stabilize USP10 or USP11 might indirectly stabilize p53 and could result in tumour suppression. On the contrary, some DUBs promote carcinogenesis (e.g. USP1, USP2a, USP6, USP8 and USP9x [56]). USP6 has been shown to be an activator of Wnt signalling by deubiquitylating the Wnt receptor Frizzled (Fzd). Deregulated Wnt signalling has implications in cancer progression. USP6 was reported to be involved in Wnt signalling, which is a key target during tumorigenesis. Discovering a deubiquitylating enzyme that can interact, stabilize or deubiquitylate USP6 is of high significance, because inhibitors against the newly identified deubiquitylating enzyme which regulates USP6 might affect USP6 protein level and its role in cancer progression. In this way, we predict an alternate indirect mechanism that might contribute to the control of tumour progression [57].

Direct targeting of the proteasome while treating solid tumours has resulted in the development of resistance against protease inhibitor, in addition to reduced efficacy and the presence of adverse effects, suggesting the need to pursue additional approaches [40,55]. For this reason, further development of novel ubiquitin proteasome system (UPS)-targeted inhibitors that can circumvent proteasome inhibitor resistance is necessary. Thus, DUBs that are known to regulate the UPS are plausible therapeutic targets. Furthermore, the DUB crystal structure provides evidence of their potential use in the treatment of diseases [51]. Although progress has been made in the development of DUB inhibitors, these inhibitors lack specificity and inhibit a wide range of DUBs [68]. To overcome this problem, extensive research on self- or trans-regulation of DUBs might significantly contribute to the understanding of the regulation of DUB expression and activity that can be modulated pharmacologically prior to inhibitor synthesis in order to apply DUBs in clinical applications.

The current conventional DUB-related therapy approach involves targeting the deubiquitylating enzymes that cause the progression of a particular disease. However, in this review, we propose an alternate viewpoint of targeting the regulators of such disease-promoting DUBs. We further hypothesize that the efficacy of a DUB inhibitor in cancers is compromised until or unless the master DUBs regulating the deubiquitylating enzyme related to disease are identified and targeted. Once the key DUBs are recognized, the task of identifying selective inhibitors can begin. Use of small molecule inhibitors seems to be an effective method to obstruct the interactions between DUBs and their substrates (other deubiquitylating enzyme). We recommend mapping of an exclusive ‘inter-DUB regulatory network’ to monitor interactions among deubiquitylating enzymes and structural characterization to identify DUBs undergoing trans-deubiquitylation. The concept of ‘dubbing DUBs’ needs to be confirmed on a wider proteomic scale to gain further insights into their mechanisms and the subsequent consequences in cell signalling and the cell cycle.

**Figure 6.**
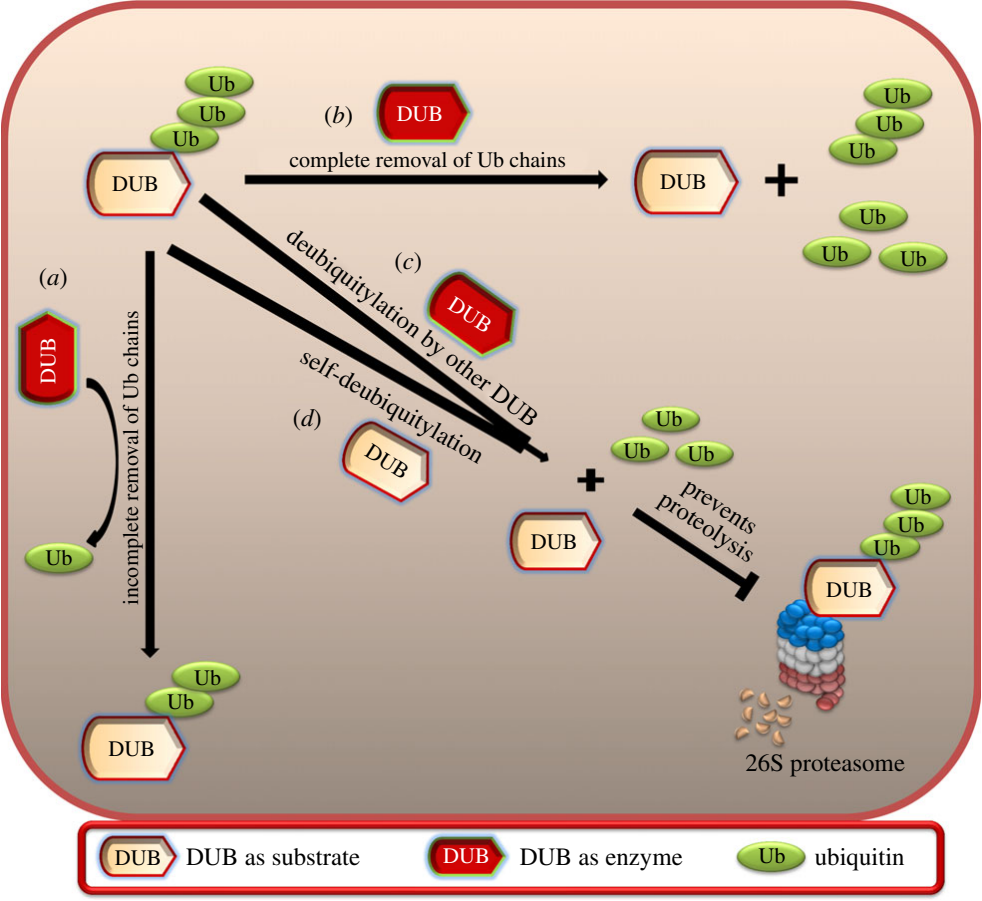
Dubbing DUBs. The figure represents DUB as a substrate as well as an enzyme and hypothetically presents the possible mechanisms of (*a*) ubiquitin chain shortening (i.e. incomplete removal of ubiquitin molecules), (*b*) ubiquitin conjugation reversal (i.e. complete removal of ubiquitin molecules), (*c*) complete deubiquitylation of ubiquitylated DUB substrate by some other DUB and (*d*) complete deubiquitylation of itself (i.e. self-deubiquitylation).

Taken together, the concept of ‘dubbing DUBs’ represents a dynamic regulatory scenario where the activity of a particular deubiquitylating enzyme is dependent on self-deubiquitylation or deubiquitylation by other members of the DUB family (figure 6). We hope that the novel ideas presented in our review might serve as useful reference for investigators concerned with deubiquitylating enzyme-targeting therapies and will result in increased knowledge about several unknown links in the DUB regulatory network. Fascinatingly, this new field of dubbing DUBs consequently might lead us to discover a novel candidate that could be a key player in the DUB signalling network. In conclusion, research in this direction would motivate the design and development of effective and specific inhibitors against the key players, which could turn out to be instrumental in the field of DUB-related therapeutics, while minimizing undesirable side effects and improving the quality of life for cancer patients.

## Data accessibility

This article has no additional data.
